# Restoration of motor learning in a mouse model of Rett syndrome following long-term treatment with a novel small-molecule activator of TrkB

**DOI:** 10.1242/dmm.044685

**Published:** 2020-11-27

**Authors:** Ian Adams, Tao Yang, Frank M. Longo, David M. Katz

**Affiliations:** 1 Department of Neurosciences, Case Western Reserve University School of Medicine, 10900 Euclid Avenue, Cleveland, OH 44106-4975, USA; 2 Department of Neurology and Neurological Sciences, Stanford University School of Medicine, Stanford, CA 94305, USA

**Keywords:** MECP2, Brain-derived neurotrophic factor, Neuro-rehabilitation, Respiration

## Abstract

Reduced expression of brain-derived neurotrophic factor (BDNF) and impaired activation of the BDNF receptor, tropomyosin receptor kinase B (TrkB; also known as Ntrk2), are thought to contribute significantly to the pathophysiology of Rett syndrome (RTT), a severe neurodevelopmental disorder caused by loss-of-function mutations in the X-linked gene encoding methyl-CpG-binding protein 2 (MeCP2). Previous studies from this and other laboratories have shown that enhancing BDNF expression and/or TrkB activation in *Mecp2*-deficient mouse models of RTT can ameliorate or reverse abnormal neurological phenotypes that mimic human RTT symptoms. The present study reports on the preclinical efficacy of a novel, small-molecule, non-peptide TrkB partial agonist, PTX-BD4-3, in heterozygous female *Mecp2* mutant mice, a well-established RTT model that recapitulates the genetic mosaicism of the human disease. PTX-BD4-3 exhibited specificity for TrkB in cell-based assays of neurotrophin receptor activation and neuronal cell survival and in *in vitro* receptor binding assays. PTX-BD4-3 also activated TrkB following systemic administration to wild-type and *Mecp2* mutant mice and was rapidly cleared from the brain and plasma with a half-life of ∼2 h. Chronic intermittent treatment of *Mecp2* mutants with a low dose of PTX-BD4-3 (5 mg/kg, intraperitoneally, once every 3 days for 8 weeks) reversed deficits in two core RTT symptom domains – respiration and motor control – and symptom rescue was maintained for at least 24 h after the last dose. Together, these data indicate that significant clinically relevant benefit can be achieved in a mouse model of RTT with a chronic intermittent, low-dose treatment paradigm targeting the neurotrophin receptor TrkB.

## Abstract (precis)

**Editor's choice:** Long-term intermittent treatment with a newly developed partial agonist of the TrkB neurotrophin receptor reverses deficits in motor learning and respiration in a mouse model of Rett syndrome.

## INTRODUCTION

Rett syndrome (RTT) is a complex neurological disorder caused by loss-of-function mutations in the gene encoding methyl-CpG-binding protein 2 (MeCP2) (Amir et al., 1999; Shahbazian and Zoghbi, 2002), a transcriptional regulatory protein (Klose and Bird, 2006). After a period of apparently normal early postnatal development, RTT patients exhibit neurological regression leading to loss of acquired speech and severe impairments in motor, cognitive, respiratory and autonomic function (Chahrour et al., 2008; Chahrour and Zoghbi, 2007; Hagberg et al., 1983; Ogier and Katz, 2008; Shahbazian and Zoghbi, 2002; Vorsanova et al., 2004; Weese-Mayer et al., 2006, 2008). In mice, inactivation of *Mecp2* at any age leads to RTT-like symptoms (Cheval et al., 2012; McGraw et al., 2011; Nguyen et al., 2012), indicating that MeCP2 protein is required across the lifespan for normal brain function. Loss of MeCP2 in RTT patients and mouse models is not associated with neuronal cell death or axonal degeneration, although neurons are smaller and more densely packed than normal, and exhibit reduced dendritic arborizations (Bauman et al., 1995), spine density and synapse number (Belichenko et al., 2009, 2008, 1997; Armstrong et al., 1995; Armstrong, 1992; Kishi and Macklis, 2010). In addition, MeCP2 deficiency results in dysregulated expression of synaptic signaling molecules (Shepherd and Katz, 2011), which, together with structural synaptic deficits, leads to abnormalities in excitatory/inhibitory synaptic balance and network connectivity (Shepherd and Katz, 2011) and, thereby, neurological dysfunction.

One of the consequences of MeCP2 deficiency that is thought to play a key role in neural circuit dysfunction in RTT is reduced activation of the tropomyosin receptor kinase B receptor (TrkB; also known as Ntrk2) by its cognate ligand, brain-derived neurotrophic factor (BDNF). BDNF–TrkB signaling appears to be disrupted in RTT by two distinct mechanisms. First, MeCP2 deficiency results in progressive, post-natal deficits in brain levels of BDNF, owing to mechanisms that remain unclear (Katz, 2014). Second, MeCP2 deficiency results in upregulation of the gene encoding protein tyrosine phosphatase 1B (PTP1B; also known as PTPN1), a transcriptional target of MeCP2 that dephosphorylates TrkB (Krishnan et al., 2015). Thus, BDNF–TrkB signaling is impaired in *Mecp2* loss-of-function mutants due to reduced BDNF availability and excessive de-phosphorylation (inactivation) of TrkB. Studies in this and other laboratories have previously shown that genetic and pharmacological approaches that enhance BDNF expression or TrkB signaling can ameliorate disease phenotypes in *Mecp2* mutant mice, including apneic breathing (Kron et al., 2014; Ogier et al., 2007; Schmid et al., 2012), motor impairments (Krishnan et al., 2015) and spatial memory deficit (Li et al., 2017).

The present study describes the detailed characterization of a second-generation, small-molecule TrkB partial agonist, PTX-BD4-3, as well as its therapeutic potential for the treatment of RTT, using heterozygous female *Mecp2* mutant mice. The primary goal of this study was to determine whether small-molecule TrkB modulation can achieve a spectrum of physiological endpoints on measures that are known to be impacted by deficits in BDNF–TrkB signaling and are relevant to clinical trial endpoints in RTT. For this purpose, we elected to use a derivative compound that would be expected to have similar ability to activate TrkB, and efficacy, as the parent compound, LM22A-4, but have a pharmacokinetic profile that is more suitable for therapeutic development. In addition, we sought to determine whether efficacy-related outcomes can be achieved with an intermittent dosing paradigm that is likely to be more attractive for clinical application than daily dosing.

## RESULTS

### Characterization of PTX-BD4-3, a second-generation, small-molecule activator of TrkB

#### PTX-BD4-3 induces biological activities via TrkB

**Fig. 1.**
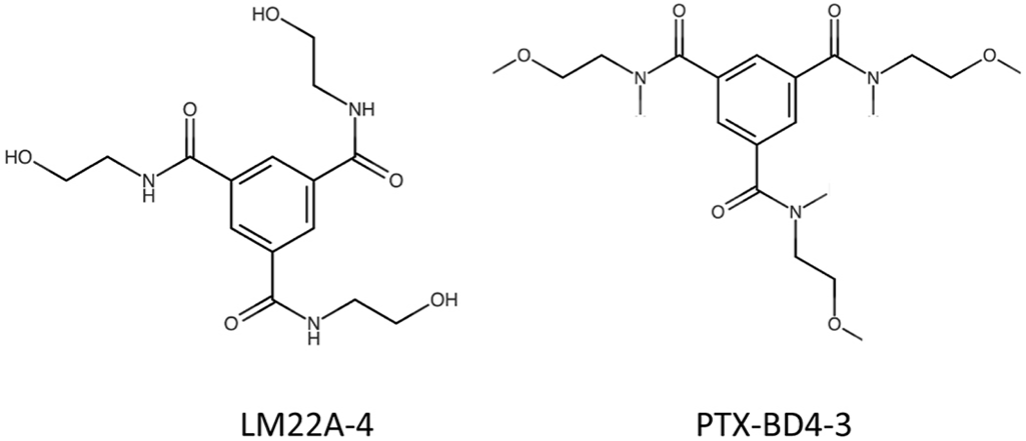
**Structure of PTX-BD4-3 and its parent compound, LM22A-4.**

The Longo laboratory previously identified LM22A-4 as a highly selective, non-peptide, small-molecule activator of TrkB (Massa et al., 2010). In an attempt to improve the potential therapeutic profile of the compound, LM22A-4 derivatives were developed with the goal of enhancing the brain-to-plasma ratio of the parent molecule. The present study reports on the detailed characterization of one such derivative, PTX-BD4-3, in which the terminal hydroxyl groups in the three side-chain moieties were substituted with methyl groups (Fig. 1), and on the *in vivo* efficacy of PTX-BD4-3 in a mouse model of RTT, a severe neurodevelopmental disorder characterized by reduced BDNF–TrkB signaling (Katz, 2014).

**Fig. 2.**
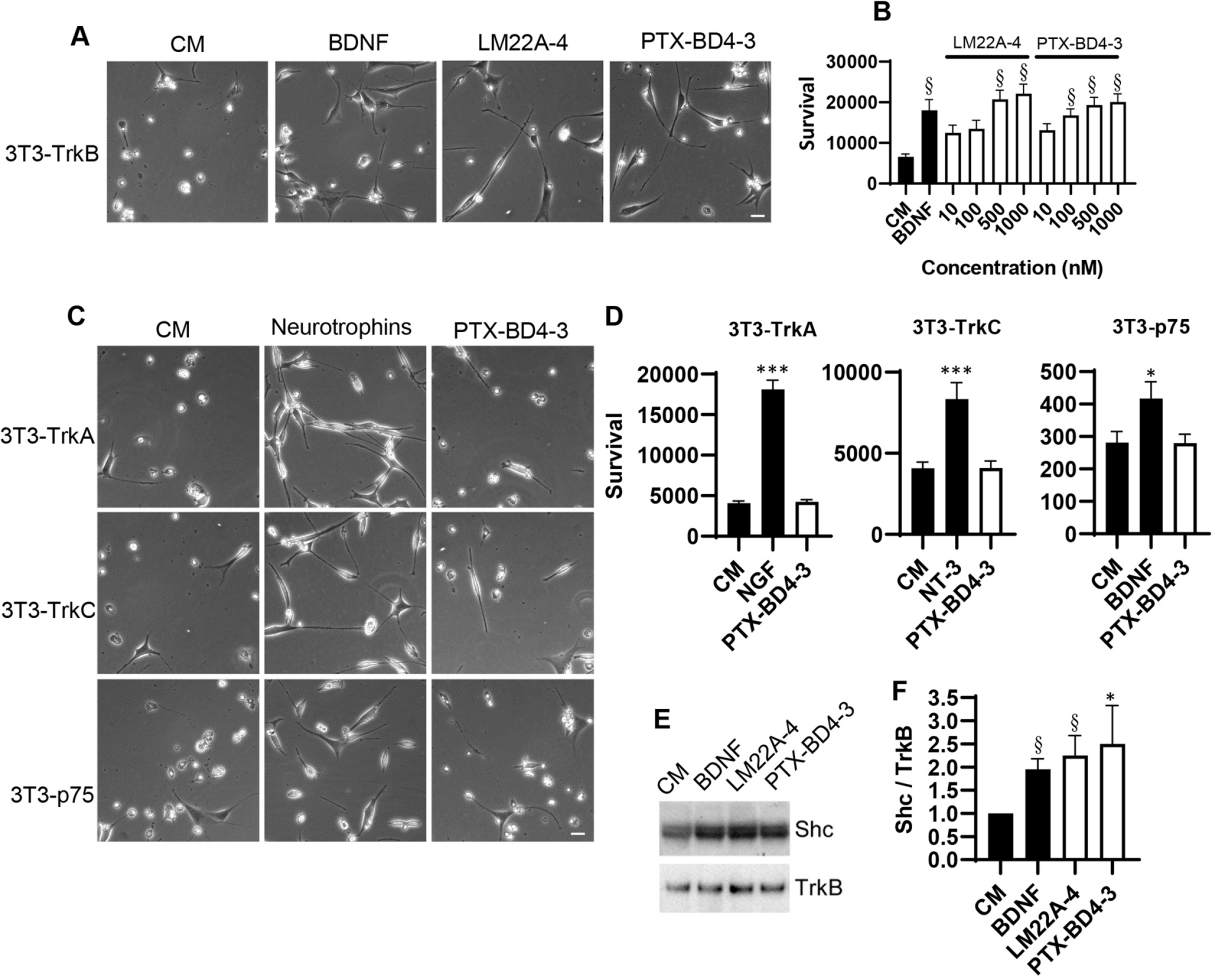
**PTX-BD4-3 promotes cell survival preferentially through TrkB. (**A-D) Neurotrophin receptor-expressing NIH-3T3 cells were incubated in culture medium (CM) alone or in medium plus either the indicated neurotrophins (BDNF, NGF, NT-3) or PTX-BD4-3 or its parent compound LM22A-4 for 72-96 h, after which survival was measured using the ViaLight assay. **(**A) Representative images showing that LM22A-4 and PTX-BD4-3 at 1000 nM support the survival of 3T3-TrkB cells. Scale bar: 10 μm. **(**B) Survival dose-response curves for PTX-BD4-3 and LM22A-4 in 3T3-TrkB cells. ^§^*P*≤0.001 compared to CM alone. (C) Representative images of 3T3-TrkA, 3T3-TrkC and 3T3-p75 cells grown in the presence of CM alone, CM+neurotrophin or CM+PTX-BD4-3. Scale bar: 10 μm. NGF, NT-3 and BDNF served as positive controls for 3T3-TrkA, 3T3-TrkC and 3T3-p75 cells, respectively. (D) PTX-BD4-3 (1000 nM) had no effect on survival of 3T3-TrkA, 3T3-TrkC or 3T3-p75 cells compared to medium alone. *P≤0.05, ***P≤0.001 compared to CM alone. For each determination in B and D, *n*=10-32 wells derived from four to five independent experiments. Statistical significance was determined by one-way ANOVA with Dunnett's post hoc multiple comparison test. (E,F) 80-100 μg/μl protein was extracted from human cementoblast-like cell (HCEM) cultures treated with either culture medium alone, BDNF, LM22A-4 or PTX-BD4-3, immunoprecipitated with anti-TrkB antibody and then probed sequentially on western blots with anti-Shc and anti-TrkB antibody. The ratio of Shc to TrkB in treated cultures was normalized and compared to control (CM alone) using Kruskal-Wallis ANOVA followed by Dunn's multiple comparisons test (*P≤0.05, ^§^*P*≤0.001). *n*=13 independent experiments, and each protein sample was run on duplicate western blots and the normalized values averaged. RLUs, relative light units.

**Fig. 3.**
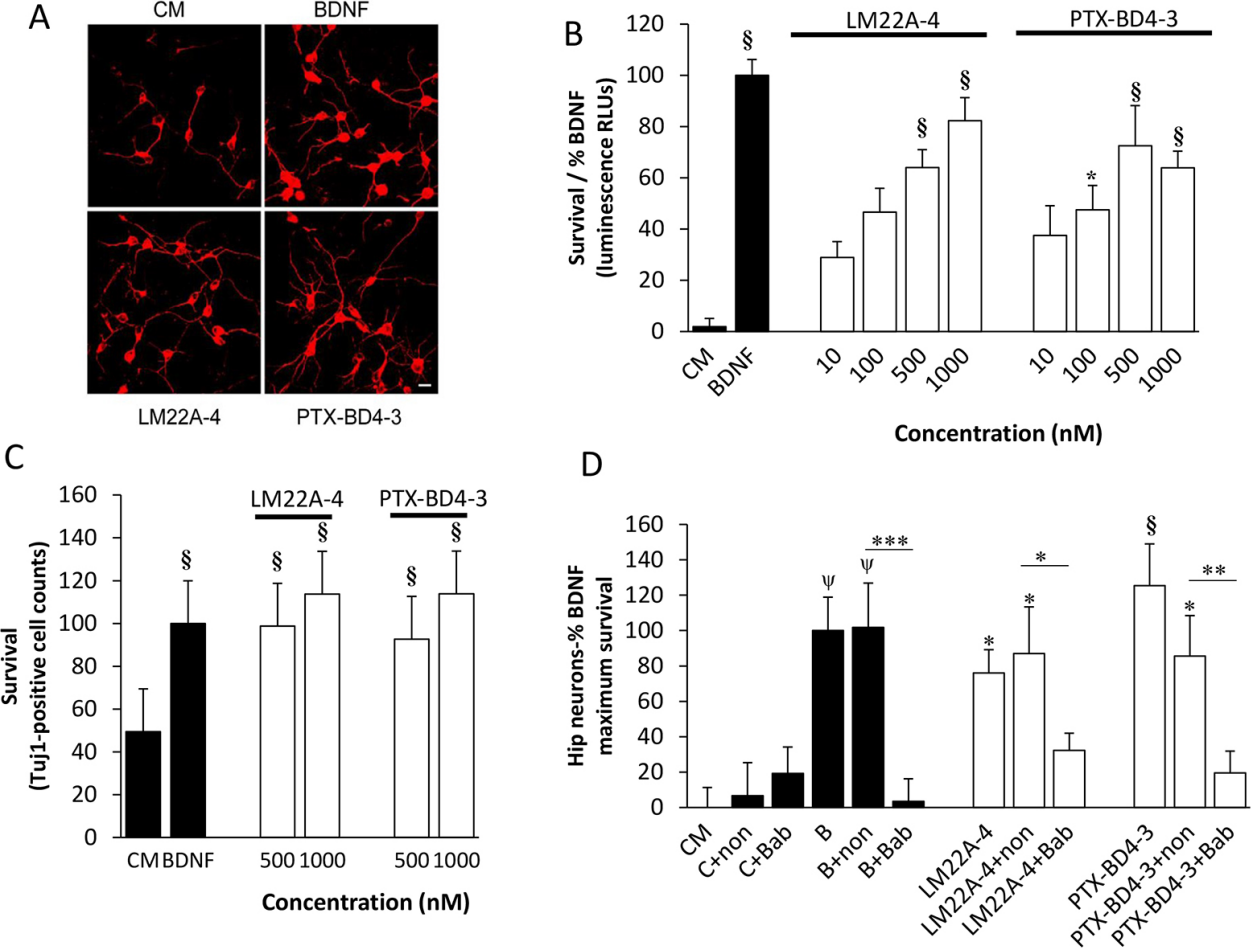
**PTX-BD4-3 acts through TrkB to support the survival of cultured hippocampal neurons.** (A) Fluorescence photomicrographs (original magnification, ×40) of Tuj1-immunostained E16 mouse hippocampal neuronal cultures treated with culture medium (CM) alone, BDNF, LM22A-4 or PTX-BD4-3 for 48 h. Scale bar: 10 μm. (B) Neuron survival dose-response curves for LM22A-4 and PTX-BD4-3 were generated using the ViaLight assay; data were normalized to survival achieved with 20 ng/ml (∼0.7 nM) BDNF and compared with wells receiving culture medium medium (CM) alone. *n*=10-32 wells per group derived from four to five independent experiments. (C) Survival analysis of hippocampal neurons treated with either 20 ng/ml BDNF, 500 nM or 1000 nM LM22A-4 or 500 nM or 1000 nM PTX-BD4-3 for 48 h. Tuj1-positive cells counts were normalized to the survival achieved with BDNF and compared with wells treated with M. *n*=32-40 wells per group derived from six independent experiments. Statistical significance in B and C was determined by one-way ANOVA with Dunnett's post hoc multiple comparison tests. *P≤0.05, ^§^*P*≤0.001 compared to control (CM). (D) Survival of hippocampal neurons treated with CM, 20ng/ml BDNF (B), 1000 nM LM22A-4 or 1000 nM PTX-BD4-3 with or without anti-TrkB antibody (Bab) or non-immune serum (non). *n*=37-42 wells per group derived from five independent experiments. *P≤0.05, **P≤0.01, ***P≤0.001 (non vs Bab), Mann-Whitney test; *P≤0.05, ^ψ^*P*≤0.01, ^§^*P*≤0.001 (compared to CM), one-way ANOVA with Dunnett's post hoc multiple comparison test.

Based on the specificity of LM22A-4 for TrkB relative to the related TrkA (also known as Ntrk1) and TrkC (also known as Ntrk3) receptors in cell survival assays (Massa et al., 2010), we first examined the survival response of 3T3 cells expressing TrkA, TrkB or TrkC to PTX-BD4-3 and exogenous neurotrophins. Adding BDNF or PTX-BD4-3 to serum-free medium did not support survival of the 3T3 parent line, which does not express Trk receptors (Soppet et al., 1991). However, cells engineered to stably express Trks exhibited robust survival responses to the cognate ligands. BDNF, NGF and NT-3 supported survival in serum-free medium of 3T3-TrkB (Fig. 2A,B), 3T3-TrkA (Fig. 2C,D) and 3T3-TrkC (Fig. 2C,D) cells, respectively. PTX-BD4-3 promoted survival of 3T3-TrkB cells in a dose-dependent manner and to a similar degree to the parent compound, LM22A-4 (Fig. 2B), but had no effect on survival of TrkA-, TrkC- or p75-expressing cells (Fig. 2D). 3T3-p75 cells did, however, respond to BDNF (Fig. 2D).

To determine whether the selectivity of PTX-BD4-3 for TrkB correlated with TrkB activation, we examined recruitment of the TrkB adaptor protein Shc (also known as Shc1) following exposure to PTX-BD4-3. Specifically, TrkB phosphorylation at Y515 promotes recruitment of Shc, which is crucial for neurotrophin-dependent cell growth and differentiation (Reichardt, 2006). Previous studies demonstrated that BDNF and LM22A-4 induced TrkB activation and Shc recruitment in human cementoblast-like cells (HCEMs) (Kajiya et al., 2014). In the present study, using the same cell system, PTX-BD4-3, like BDNF and its parent compound LM22A-4, promoted Shc recruitment to TrkB (Fig. 2C), confirming that PTX-BD4-3 triggers activation of TrkB.

To further confirm that PTX-BD4-3 promotes biologically relevant activity through TrkB, we examined the effect of PTX-BD4-3 on survival of cultured hippocampal neurons, using two different methods (Fig. 3A-C). Fig. 3B shows survival estimates derived from measurements of ATP concentration in hippocampal neuron cultures (ViaLight assay), which is considered a measure of basic mitochondrial function and widely used as a surrogate measure of cell viability. As a second approach, Fig. 3C shows counts of β-tubulin III (Tuj1)-stained neurons, a separate and also widely used method for assessment of neuronal survival. It would be expected that although there is a correlation between survival status and mitochondrial metabolic status, one could have some degree of variability in metabolic function for a given level of cell survival. In both sets of studies, neurotrophic activity dose-response studies with PTX-BD4-3 demonstrated maximum levels of activity in the range of 80-100% of that of BDNF, half-maximal effective concentration (EC50) values of 300-500 nM, and efficacy similar to that of the parent compound, LM22A-4 (Fig. 3A-C). Because the plateau effect of PTX-BD4-3 on survival was observed between 500 nM and 1000 nM, we chose 1000 nM as a standard concentration for subsequent mechanistic studies.

To independently test and further confirm the necessity of TrkB activation in the biological activity of PTX-BD4-3, we examined the effects of a function-blocking antibody directed against the extracellular domain (ECD) of TrkB (anti-TrkB^ECD^) that is known to inhibit BDNF activity (Massa et al., 2010). Treatment of hippocampal neuron cultures with anti-TrkB^ECD^, but not control antibody, led to a reduction in the neurotrophic activity of BDNF, LM22A-4 and PTX-BD4-3 (Fig. 3D), supporting the necessity of TrkB engagement for PTX-BD4-3 biological activity.

To further define the specificity of PTX-BD4-3 for TrkB, we screened 55 pharmacologically relevant receptors, including neurotransmitter and G-protein coupled receptors for PTX-BD4-3 binding (Table S1). Within this screen, performed by Cerep, Inc. (France), inhibition of binding by a test ligand of greater than 50% is considered to demonstrate significant binding by the test compound. At the standard screening concentration of 10 μM, PTX-BD4-3 demonstrated no significant binding within the panel, similar to its parent compound LM22A-4 (Massa et al., 2010). Thus, as with LM22A-4 (Massa et al., 2010), the specificity of PTX-BD4-3 for TrkB is supported by multiple lines of evidence, including receptor binding, receptor activation, receptor adaptor recruitment and cell survival assays.

#### Brain and plasma levels and *in vitro* ADME-Tox analysis

To gain additional insight into the potential suitability of PTX-BD4-3 as a candidate lead molecule, we compared brain and plasma levels of the compound and its parent, LM22A-4, by reverse-phase liquid chromatography with triple-quadrupole tandem mass spectroscopic detection (LC-MS/MS) following intraperitoneal (i.p.) administration in mice (Fig. S1). We specifically tested the hypothesis that methyl substitutions at the terminal hydroxyl groups in the three side-chain moieties would increase the brain-to-plasma ratio of PTX-BD4-3 compared to the parent compound. At 1 h and 3 h after i.p. administration of 50 mg/kg of each compound, brain levels (nM) of PTX-BD4-3 and LM22A-4 were not significantly different from each other [1 h: PTX-BD4-3, 227.2942; LM22A-4, 199.7788 (*P*=0.202); 3 h: PTX-BD4-3, 36.05238; LM22A-4, 53.16617 (*P*=0.380)]. Similarly, at 1 h after injection, plasma levels of PTX-BD4-3 and LM22A-4 were not significantly different [PTX-BD4-3, 10011.16; LM22A-4, 9193.46 (*P*=0.167)] and the brain-to-plasma ratios were comparable [PTX-BD4-3, 0.03294; LM22A-4, 0.02681 (*P*=0.139)]. However, at 3 h after administration, the plasma level of PTX-BD4-3 was significantly lower than that of LM22A-4 [PTX-BD4-3, 361.446 µM; LM22A-4, 2349.843 µM (*P*=0.030)], resulting in a higher brain-to-plasma ratio for PTX-BD4-3 [PTX-BD4-3, 0.271; LM22A-4, 0.133 (*P*=0.038)]. In addition, these experiments indicated that the brain level of PTX-BD4-3 at 1 h after systemic administration was well within the concentration range that promotes neurotrophic activity in assays with 3T3 cells (Fig. 2) and hippocampal neurons (Fig. 3), and that the half-life of the compound in both brain and plasma is less than 3 h.

*In vitro* absorption, distribution, metabolism, excretion and toxicity (ADME-Tox) analysis (Cerep, Inc.) indicated that PTX-BD4-3 had no activity in assays of DNA mutagenesis or cardiac toxicity and was negative for cytochrome P450 (CYP) inhibition (Table S2).

### PTX-BD4-3 activates TrkB signaling *in vivo*

**Fig. 4.**
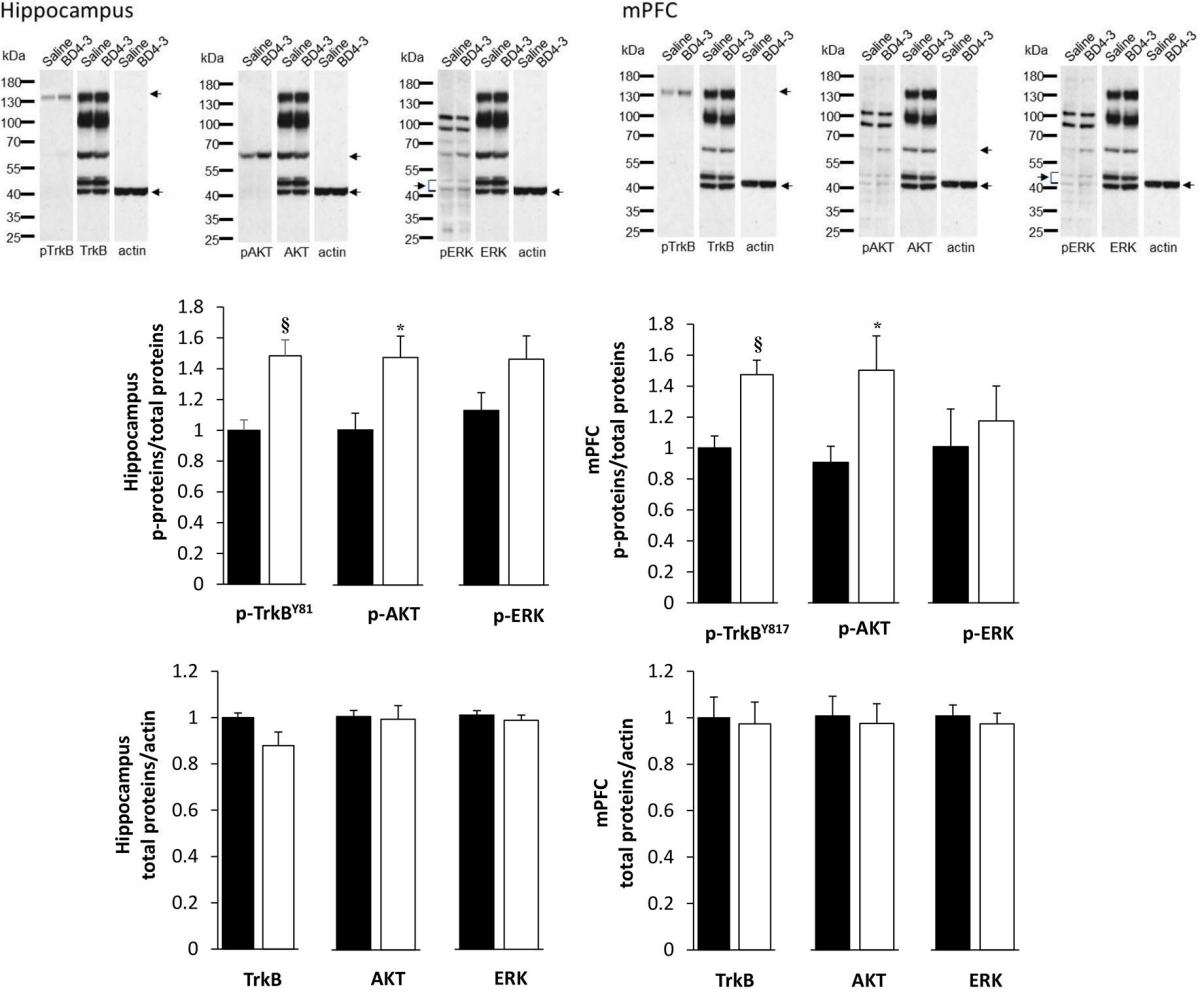
**PTX-BD4-3 activates TrkB signaling *in vivo* following systemic administration in null male *Mecp2* mutant mice.** Null mice were treated for 5 days with once daily injections of PTX-BD4-3 (open bars, 50 mg/kg, i.p.) or saline (black bars, vehicle) and then sacrificed 1 h after the last injection. Brain lysates from the hippocampus and medial prefrontal cortex (mPFC) were processed for western blot analysis of phosphorylated and total TrkB (pTrkB^Y817^/TrkB), AKT (pAKT/AKT) and ERK (pERK/ERK) as described in the Materials and Methods. For detection of total TrkB, AKT and ERK, blots were concomitantly probed with the respective total protein antibodies and each of the three bands quantitated as indicated by arrows. For ERK, the pair of bands (consistent with ERK1 and ERK2 isoforms) indicated by the brackets was quantitated together as one value. For each tissue, the same blot and images for total protein (TrkB, AKT, ERK, actin) are shown here in triplicate as an adjacent reference to phospho-protein detection. For detection of phospho-proteins in the hippocampus, blots were probed separately with the three indicated phospho antibodies. For detection in the mPFC, blots were probed separately for pTrkB and probed for pAKT and pERK concomitantly; and for the latter two, the same blot and image are shown here in duplicate as an adjacent reference to phospho-protein detection. **^§^***P*<0.01 (compared to vehicle); *P<0.05 (compared to vehicle); Student's *t*-test.

In light of our finding that PTX-BD4-3 exhibits robust activity in assays of TrkB function *in vitro*, we next examined whether or not PTX-BD4-3 activates TrkB signaling *in vivo* following systemic administration in mice. To approach this issue we used hemizygous male *Mecp2* null mice, a model of RTT that exhibits postnatal deficiencies in brain levels of BDNF as well as reduced TrkB signaling (Katz, 2014; Li and Pozzo-Miller, 2014). Animals were given either single or repeated doses of PTX-BD4-3 (50 mg/kg, i.p.) or vehicle (saline) and then sacrificed for tissue harvesting and western blot analysis of phosphorylated and total TrkB (pTrkB/TrkB), AKT (also known as AKT1) (pAKT/AKT) and ERK (also known as MAPK) (pERK/ERK), respectively, in the hippocampus and medial prefrontal cortex (mPFC). Treatment with PTX-BD4-3 significantly increased pTrkB/TrkB and pAKT/AKT, and had no significant effect on pERK/ERK (Fig. 4). No differences were observed in expression of total TrkB, AKT or ERK between saline- and PTX-BD4-3-treated animals (Fig. 4).

### Efficacy of PTX-BD4-3 in *Mecp2* mutant mice

#### Symptom reversal following chronic dosing of heterozygous female *Mecp2* mice with PTX-BD4-3

In light of evidence that deficits in BDNF expression and TrkB signaling contribute to neurological dysfunction in mouse models of RTT (Katz, 2014), we next examined whether or not chronic treatment of *Mecp2* mutant mice with PTX-BD4-3 would ameliorate disease phenotypes. For these studies, we used heterozygous (Het) female *Mecp2* mutants, which, unlike male null mice, model the somatic mosaicism for normal and mutant MeCP2 that characterizes the human disease (Katz et al., 2012). Animals were treated once every 3 days (5 mg/kg, i.p.), beginning at 16 weeks of age, and neurological phenotypes were assessed at various time points up to 8 weeks of dosing. All tests were performed 24 h after the last treatment. Phenotypic assessments included whole-body plethysmography to quantify apneic breathing, a core feature of respiratory dysfunction in RTT, as well as a motor test battery. Chronic intermittent dosing with PTX-BD4-3 was well tolerated, as body weight was unchanged compared to that of saline-treated Het control littermates (Fig. S2). In fact, unlike saline-treated Het animals, the global phenotypic severity scores of animals treated with PTX-BD4-3 did not increase significantly over the time course of the experiments (Table S3).

##### Respiratory dysfunction

**Fig. 5.**
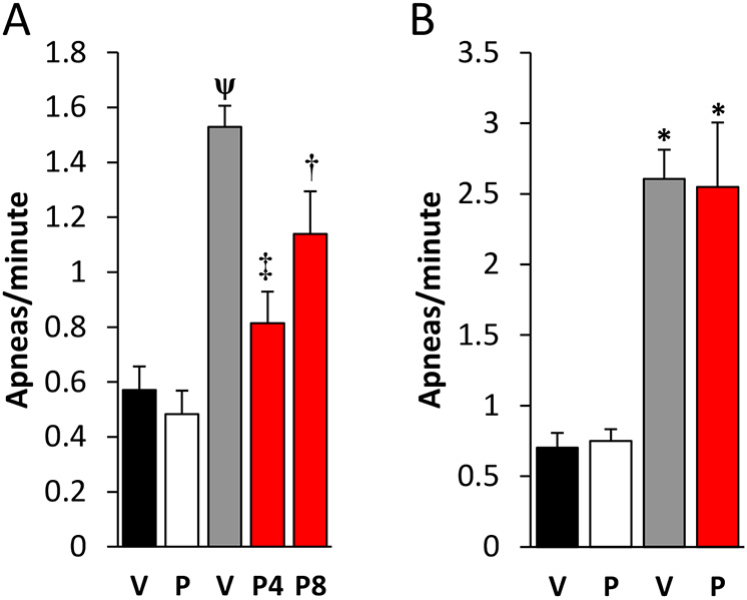
**Chronic intermittent dosing with PTX-BD4-3 reduces apneic breathing in heterozygous (Het) female *Mecp2* mutants.** (A) Repeated dosing with PTX-BD4-3 [P; 5 mg/kg, once every 3 days (q72 h)] for either 4 (P4) or 8 (P8) weeks results in a durable reduction in the apnea index in Het mice compared to saline vehicle (V), measured 24 h after the last dose. *n*=10-37 subjects derived from two to four independent experiments. (B) Treatment of 24-week-old mice with a single dose of PTX-BD4-3 (5 mg/kg) has no effect on the apnea index, measured 24 h after dosing. *n*=6-12 subjects derived from two independent experiments. *P<0.05, **^ψ^***P*<0.001 compared to vehicle-treated Wt mice; ^†^*P*<0.05, ^‡^*P*<0.001 compared to saline-treated Het mice. One-way ANOVA with Bonferroni post hoc analysis.

As previously described, Het mice exhibited significantly more respiratory apneas (defined as breaths lasting longer than 2× the average total breath duration; Ttot) than wild-type (Wt) mice. However, after 4 and 8 weeks of repeated intermittent dosing with PTX-BD4-3, the apnea index in Het mice, measured 24 h after the last dose, was significantly reduced compared to that in saline-treated Het littermates (Fig. 5A). To determine whether or not the apnea rescue observed after chronic intermittent treatment with PTX-BD4-3 was, in fact, due to repeated dosing or simply an effect of the last drug dose, we also measured the apnea index 24 h after a single dose of PTX-BD4-3 (5 mg/kg, i.p.) administered to drug-naïve Wt and Het mice at 24 weeks of age, corresponding to the age of the animals at the end of the 8-week chronic dosing protocol. In contrast to animals that received chronic dosing, we saw no change in the apnea index in response to single dosing at 24 weeks of age (Fig. 5B).

##### Motor dysfunction

**Fig. 6.**
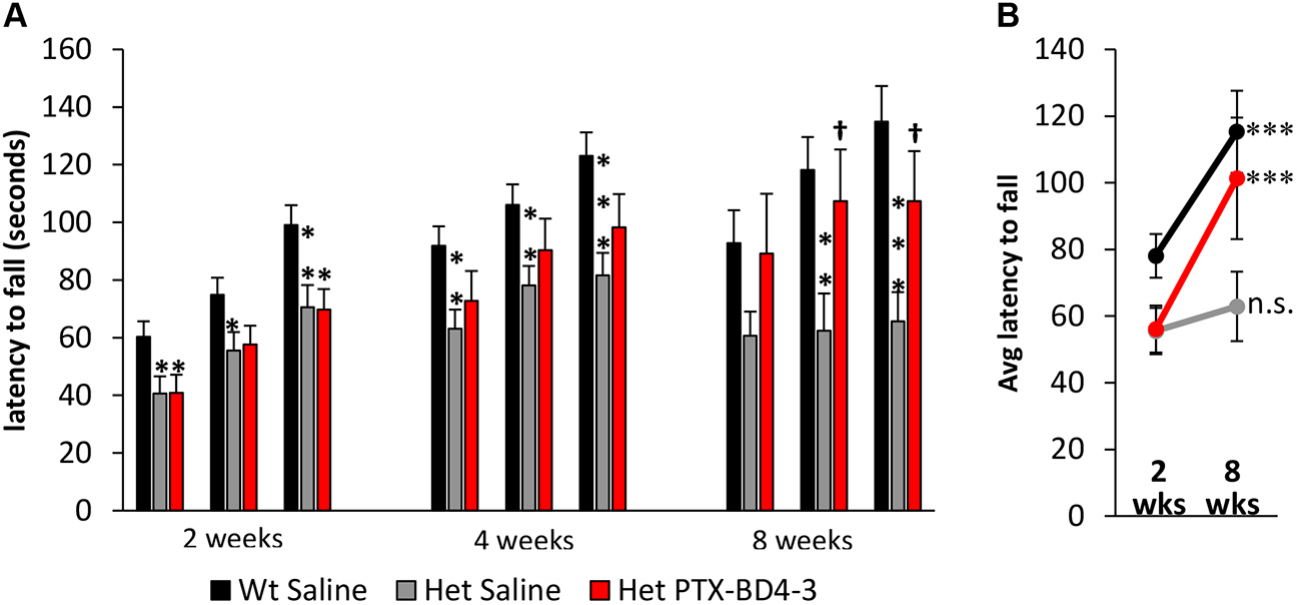
**Chronic intermittent dosing with PTX-BD4-3 restores motor learning in Het female *Mecp2* mutants.** (A) Repeated dosing with PTX-BD4-3 (5 mg/kg, q72 h) for 2, 4 and 8 weeks results in a progressive improvement in Rotarod performance (latency to fall) in Het mice, measured 24 h after the last dose. Rotarod performance was measured over three successive trials at each time point. *P<0.05 compared to saline-treated Wt mice (Wt Saline), ^†^*P*<0.05 compared to saline-treated Het mice (Het Saline); one-way ANOVA with LSD post hoc analysis. (B) Comparison of Rotarod performance, averaged across all three trials after 2 and 8 weeks of treatment, illustrates that Het animals treated with PTX-BD4-3 showed significantly greater improvement over time than their saline-treated Het littermates. ***P<0.001 compared to 2-week value, unpaired Student's *t*-test; n.s., not significant compared to 2-week value. *n*=14-37 subjects derived from two to three independent experiments.

**Fig. 7.**
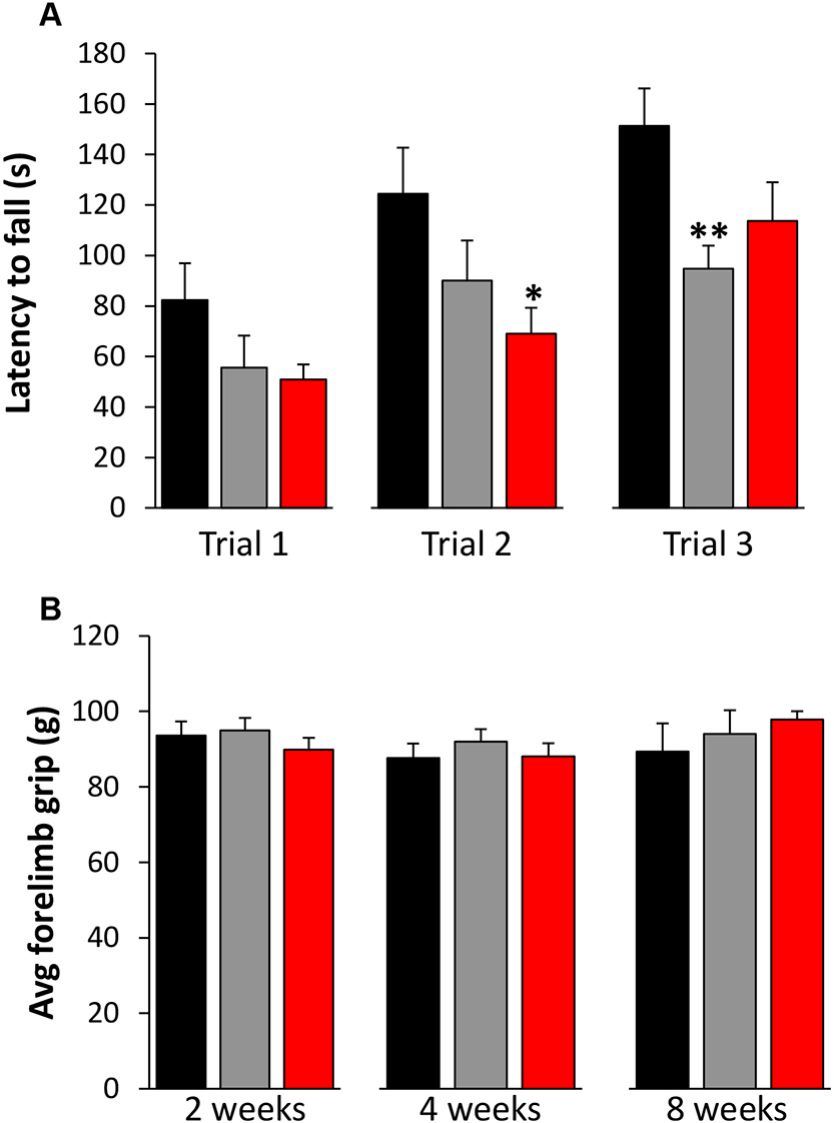
**Chronic intermittent dosing with PTX-BD4-3 has no effect on Rotarod performance in heterozygous (Het) female *Mecp2* mutants that did not receive prior Rotarod training during the treatment period.** (A) Repeated dosing with PTX-BD4-3 (5 mg/kg, q72 h) for 8 weeks had no impact on latency to fall in animals that were tested only at the end of the 8-week treatment period. Rotarod performance was measured over three successive trials, 24 h after the last dose. *n*=15-16 subjects derived from two independent experiments. (B) Het mice show no deficit in forelimb grip strength compared to Wt controls, and no increase in grip strength in response to treatment with PTX-BD4-3. *n*=9-20 subjects derived from two independent experiments. *P<0.05, **P<0.001 compared to saline-treated Wt mice. One-way ANOVA with LSD post hoc analysis. Black, saline-treated Wt mice; gray, saline-treated Het mice; red, PTX-BD4-3-treated Het mice.

Over the course of the 8-week treatment period, which included three Rotarod performance assays (at 2, 4 and 8 weeks of treatment), consisting of three trials each, Wt mice treated with saline showed significant improvement in Rotarod performance, i.e. increased latency to fall. This improvement is consistent with previous reports of motor learning in response to repeated exposure to Rotarod testing (Buitrago et al., 2004). However, Het mice treated with saline exhibited significantly shorter latencies to fall compared to Wt littermates at each of the three time points tested (2, 4 and 8 weeks of treatment) and showed no improvement over time (Fig. 6A,B). In contrast, although Het mice treated with PTX-BD4-3 were as impaired as their saline-treated Het littermates at the 2-week time point, their performance progressively improved, and by 8 weeks of treatment they performed at a level that was comparable to that of saline-treated Wt controls (Fig. 6A,B). The fact that Het mice treated with PTX-BD4-3 exhibited a Wt-like learning curve suggested that the treatment was impacting motor learning in response to repeated Rotarod testing, rather than, or in addition to, other components of motor function, such as grip strength. To investigate this possibility, we evaluated Rotarod performance in additional cohorts of animals that received chronic intermittent dosing with PTX-BD4-3 and only one exposure to Rotarod testing, at the end of the 8-week treatment period (Fig. 7A). In contrast to animals that were exposed to repeated Rotarod testing throughout the treatment period, animals tested only at the end of the treatment period showed no improvement in performance compared to saline-treated Het controls. Moreover, Het animals showed no deficit in forelimb grip strength compared to Wt, and no gain in grip strength after treatment with PTX-BD4-3 (Fig. 7B).

In contrast to the marked improvement in Rotarod performance, Het mice treated chronically with PTX-BD4-3 exhibited no reduction in foot slip errors measured 24 h after the last treatment (data not shown). Notably, in a pilot study on acute effects on motor performance, we observed a dose-dependent reduction in foot slip errors measured 1 h after a single injection of PTX-BD4-3 (50 mg/kg, i.p.) compared to saline-treated Het controls (Fig. S3).

## DISCUSSION

The present findings demonstrate that a low-dose, chronic intermittent treatment paradigm targeting the neurotrophin receptor TrkB can yield significant, clinically relevant symptomatic benefit in a mouse model of RTT. We used a novel, second-generation, small-molecule activator of TrkB, PTX-BD4-3, that was derived from LM22A-4, a first-in-class TrkB partial agonist that has been shown to activate TrkB in diverse *in vitro* and *in vivo* systems (Massa et al., 2010; Han et al., 2012; Simmons et al., 2013; Schmid et al., 2012; Al-Qudah et al., 2014; Kajiya et al., 2014; Yu and Wang, 2015; Nomura et al., 2017; Li et al., 2017; Pedard et al., 2018; Geraghty et al., 2019; Nguyen et al., 2019), although some *in vitro* assays have not shown activity (Todd et al., 2014; Boltaev et al., 2017). In *in vitro* assays, PTX-BD4-3 had similar potency and efficacy to the LM22A-4 parent compound. In addition, PTX-BD4-3 exhibited a higher brain-to-plasma ratio at 3 h after administration, possibly due to more rapid clearance from the blood compared to LM22A-4 (Fig. S1). Most importantly, higher drug brain-to-plasma ratios reduce risks for peripheral toxicity, and our findings warrant a more detailed characterization of the pharmacokinetic profile of PTX-BD4-3, including brain tissue bioavailability studies, in subsequent studies. In addition, it is important to note that specificity for TrkB relative to TrkA and TrkC was maintained in PTX-BD4-3. In addition to its receptor-specific and receptor-dependent biological activity in cell-based assays of TrkB function, along with the ability of TrkB extracellular domain antibodies to block its function, we also demonstrated the ability of PTX-BD4-3 to promote recruitment of the Shc adaptor protein to TrkB *in vitro* and to induce phosphorylation of TrkB and activation of downstream signaling *in vivo*. These four lines of evidence strongly support a model in which PTX-BD4-3 functions through activation of TrkB.

In addition to supporting the hypothesis that reduced BDNF–TrkB signaling contributes to neurologic dysfunction in RTT, our data suggest that, despite MeCP2 deficiency, repetitive stimulation of TrkB over a prolonged period of time can produce meaningful functional plasticity and symptom improvement that is not seen with single dosing. For example, whereas Het mice treated intermittently with PTX-BD4-3 from 16 to 24 weeks of age demonstrated a significant reduction in apneic breathing, there was no change in the apnea index in drug-naïve animals treated with a single dose of PTX-BD4-3 at 24 weeks of age. These data may indicate that single and repeated dosing engage distinct mechanisms of TrkB signaling. For example, transient and sustained activation of TrkB, respectively, have previously been shown to elicit distinct profiles of downstream signaling (Guo et al., 2018), in some cases persisting over hours and days. Alternatively, the underlying mechanism(s) may be the same and simply more effectively engaged by repeated dosing. In either case, given the short brain half-life of PTX-BD4-3 (Fig. S1), the fact that treated Het mice exhibit symptom recovery 24 h after the last dose indicates that the benefits of chronic intermittent dosing persist for some time after PTX-BD4-3 has been cleared from the brain. This long time course of effects after intermittent dosing is consistent with the fact that even transient TrkB activation leads to downstream sequelae with relatively long time constants, including changes in gene expression, neurite elongation and dendritic spine growth (Guo et al., 2018).

These findings are consistent with previous findings of improvements in RTT mouse models following treatments that either increase BDNF levels (Chang et al., 2006; Ogier et al., 2007; Deogracias et al., 2012), activate TrkB (Schmid et al., 2012; Kron et al., 2014; Li et al., 2017; Johnson et al., 2012) or reduce TrkB dephosphorylation (Krishnan et al., 2015). However, this is, to our knowledge, the first demonstration that pharmacological treatment targeting the BDNF–TrkB signaling pathway can restore motor learning in an RTT model, although the parent compound, LM22A-4, has been shown to improve motor learning in a rat model of traumatic brain injury (Massa et al., 2010). Over the course of the 8-week treatment period, which included three Rotarod performance assays consisting of three trials each, Wt mice treated with saline showed significant improvement in Rotarod performance, i.e. increased latency to fall. This improvement is consistent with previous studies demonstrating the importance of motor learning in the gains in Rotarod performance that are observed following repeated test exposures in rodents (Buitrago et al., 2004). In contrast, during this same period, Het mice treated with saline showed no improvement. However, Het mice treated with PTX-BD4-3 exhibited a marked increase in latency to fall over the course of treatment, and, by the 8-week time point, their performance was not significantly different from that of Wt animals. To determine whether or not this improvement reflected an effect of TrkB activation on motor learning in response to the multiple Rotarod test exposures, we also examined the effects of chronic intermittent treatment with PTX-BD4-3 in Het animals that were tested on the Rotarod only once, at the end of the 8-week treatment period. In this case, PTX-BD4-3 had no effect on latency to fall, consistent with the hypothesis that TrkB activation improves Rotarod performance in Het mice in the context of motor learning. This hypothesis is further supported by the fact that, whereas Het mice treated with saline showed a complete flattening of their within-trial learning curve by the 8-week time point, this is less apparent in Het mice treated with PTX-BD4-3 (Fig. 7A). Our finding that treatment with PTX-BD4-3 improves Rotarod motor learning in Het mice is consistent with previous studies demonstrating improved motor learning following exposure of RTT mice to enriched environments, a manipulation that can increase BDNF expression in some brain regions (Kondo et al., 2008; Lonetti et al., 2010). More generally, our data are consistent with earlier studies demonstrating a role for BDNF–TrkB signaling in use-dependent plasticity of motor learning circuits in normal animals. Rotarod motor learning is associated with mechanisms of cerebellar plasticity, such as decreased mossy fiber synapse density (Ruediger et al., 2011), that are regulated by BDNF–TrkB signaling (Rico et al., 2002; Chen et al., 2016). More recently, BDNF induction in layer II/III neurons in motor cortex has been shown to be essential for motor learning in mice (Andreska et al., 2020). Moreover, the *BDNF*^Met/Met^ allele, which impairs activity-dependent BDNF secretion (Chen et al., 2006), is associated with decreased Rotarod motor learning in mice (Fritsch et al., 2010) and motor learning deficits in humans (Fritsch et al., 2010). There is also evidence that TrkB signaling is important for non-motor learning, including in RTT mice. For example, long-term treatment of Het mice with LM22A-4 improves behavioral learning in a spatial memory task (Li et al., 2017). The fact that exposure of Het mice to PTX-BD4-3 during Rotarod testing was associated with subsequent restoration of motor learning raises the possibility that the effectiveness of motor learning strategies in RTT patients, e.g. in the context of neuro-rehabilitative therapy, might be improved by concomitant administration of a pharmacologic activator of TrkB.

Chronic intermittent dosing with PTX-BD4-3 also resulted in a significant reduction in the apnea index in Het mice. Identifying specific circuit mechanisms or anatomic loci at which PTX-BD4-3 acts to ameliorate the apnea phenotype is complicated by the multiplicity of neuronal targets at which MeCP2 deficiency, as well as impaired BDNF–TrkB signaling, may impact apneic breathing. Previous studies have identified multiple sites within the central neuraxis in *Mecp2* mutants where reduced BDNF expression can impact respiratory motor output and where TrkB activation could act to improve respiratory function, including the ponto-medullary respiratory network and suprabulbar structures that modulate brainstem respiratory output (Katz, 2014) Data from numerous laboratories indicate that MeCP2 deficiency is associated with increased excitability within brainstem cell groups involved in the generation and modulation of the respiratory pattern, including the preBotzinger complex, and the Koelliker-Fuse, locus coeruleus and solitary tract nuclei (nTS), respectively (Stettner et al., 2007; Kline et al., 2010; Taneja et al., 2009; Ramirez et al., 2013). Findings of respiratory hyperreflexia in *Mecp2* mutant mice (Roux et al., 2008; Voituron et al., 2009) are also consistent with brainstem hyperexcitability, particularly within respiratory sensory relay nuclei such as the nTS. Given our previous findings that BDNF and LM22A-4 reduce synaptic hyperexcitability in nTS in isolated brainstem preparations, one possibility is that PTX-BD4-3 reduces apneas by reducing synaptic hyperexcitability at the level of the brainstem (Kline et al., 2010; Kron et al., 2014). However, the brainstem respiratory network also receives strong modulatory inputs from forebrain structures, including the mPFC and amygdala, which play crucial roles in behavioral state-dependent changes in respiratory output and are significantly impacted by MeCP2 deficiency (Howell et al., 2017). In the mPFC, for example, loss of MeCP2 is associated with hypoactivity of pyramidal neurons due to reduced excitatory synaptic drive (Sceniak et al., 2015). Moreover, chemogenetic activation of pyramidal neurons in the MeCP2-deficient mPFC eliminates apneas through a mechanism that appears to involve a reduction in brainstem hyperexcitability (Howell et al., 2017). Given that BDNF/TrkB activation can enhance excitatory synaptic connectivity in the mPFC (Fukumoto et al., 2019), it is therefore possible that PTX-BD4-3 treatment reduces apneic breathing by increasing activity in descending modulatory inputs from the mPFC to the brainstem respiratory network, in addition to potential direct actions within the brainstem itself.

Overall, the profile resulting from the *in vitro* ADME-Tox analysis (see Tables S1 and S2), along with the evidence of marked efficacy in *Mecp2* mutant mice, indicates that PTX-BD4-3 can be considered as a candidate for further characterization, including formal investigational new drug studies for potential clinical trials, in the context of RTT.

## MATERIALS AND METHODS

### Animals

Studies on CF1 mice were performed in the Longo laboratory. Embryonic day (E)16 timed pregnant mice were purchased from Charles River (Hollister, CA, USA) and sacrificed immediately upon delivery to the Longo laboratory. All experimental procedures were approved by and conducted in accordance with the guidelines of the Institutional Animal Care and Use Committee (IACUC) at Stanford University. Studies on *Mecp2* mutant mice were performed in the Katz laboratory. These studies were conducted with Wt and Het littermates from the Katz laboratory breeding colony of *Mecp2^tm1.1Jae^* mice, originally developed by Dr R. Jaenisch (Whitehead Institute, Massachusetts Institute of Technology, Cambridge, MA, USA) and purchased from the Mutant Mouse Regional Resource Center (University of California Davis, Davis, CA, USA). Mice were maintained on a mixed genetic background (129Sv, C57BL/6, BALB/c) by non-sibling crosses of Hets (*Mecp2^tm1.1Jae^*^/+^) with Wt males from a separate cohort of *Mecp2^tm1.1Jae^* (129Sv, C57BL/6, BALB/c) mice. All experimental procedures were approved by and conducted in accordance with the guidelines of the IACUC at Case Western Reserve University.

### Reagents

Recombinant BDNF was purchased from PeproTech (Rocky Hill, NJ, USA). LM22A-4 [N,N′,N′′-tris(2-hydroxyethyl)-1,3,5-benzenetricarboxamide] and PTX-BD4-3 {benzene-1,3,5-tricarboxylic acid tris-[(2-methoxy-ethyl)-methyl-amide]} were custom manufactured by Ricerca Biosciences (Concord, OH, USA). Each preparation was characterized by high-performance liquid chromatography and liquid chromatography/mass spectrometry, and had a purity of greater than 97%. The molecular mass and formula of the compounds were further confirmed by high-resolution mass spectrometry. Other reagents were purchased from Sigma-Aldrich (St Louis, MO, USA) unless otherwise stated.

NIH-3T3 cell cultures and cell survival assays

Mouse NIH-3T3 cells expressing TrkA (NIH-3T3-TrkA) were provided by Dr William Mobley (University of California at San Diego, San Diego, CA, USA), and NIH-3T3 cells expressing TrkB (NIH-3T3-TrkB) or TrkC (NIH-3T3-TrkC) were provided by Dr David Kaplan (University of Toronto, Toronto, ON, Canada). Cells were propagated in Dulbecco's modified Eagle medium (DMEM) supplemented with 10% fetal bovine serum (FBS; Invitrogen) and 200-400 µg/ml Geneticin. Cells were seeded into 24-well plates (30,000 cells/well) and cultured in medium consisting of 50% PBS and 50% DMEM without supplements. Following exposure to BDNF (20 ng/ml, 0.7 nM) or 10-1000 nM LM22A-4 or PTX-BD4-3 for 72-96 h, cells were suspended in 50 µl lysis buffer, transferred to white, opaque 96-well culture plates and survival was measured using the ViaLight Assay (Lonza Group, Rockland, ME, USA).

### Primary neuronal cultures and cell survival assays

Hippocampal neuron cultures were prepared from E16 CF1 mouse fetuses as described previously (Yang et al., 2006). Under the low-density conditions used here, neuronal survival is dependent, in part, on addition of exogenous neurotrophins (Yang et al., 2008; Walter et al., 2011). LM22A-4 and PTX-BD4-3 were dissolved in water at a stock concentration of 10 mM prior to dilution (1:10,000) in culture medium. Cell survival following a 48-h exposure to BDNF, LM22A-4 or PTX-BD4-3 was quantified by either counting β-tubulin III-positive cell bodies (Massa et al., 2006; Walter et al., 2011) or by the ViaLight Assay described above.

### Shc adaptor protein assays

HCEMs (a gift from Dr Kitagawa, Hiroshima University Hospital, Hiroshima, Japan) stably transfected with the telomerase catalytic subunit *TERT* gene, were cultured in α-minimum essential medium (α-MEM, Sigma-Aldrich) supplemented with 10% FBS to 80-90% confluence. Prior to the addition of ligands, the medium was changed to α-MEM with 0.5% FBS, and cells were incubated for 4 h and then treated with BDNF (0.7 nM) or LM22A-4 (1000 nM) or PTX-BD4-3 (1000 nM) for 1 h. Cells were then lysed, and 80-100 µg/µl extracted protein was immunoprecipitated with anti-TrkB antibody (1:100; 07-225, Millipore); the precipitated protein was then retained for western blot analyses using anti-Shc antibody (1:1000; 2432, Cell Signaling Technology, Beverly, MA, USA) or anti-TrkB antibody (1:3000, 07-225, Upstate USA, Charlottesville, VA, USA). To detect the immunoblotted target protein bands without interference from denatured IgG, the horseradish peroxidase (HRP)-conjugated anti-rabbit IgG VeriBlot for IP secondary antibody (1:1000; ab131366, Abcam, Cambridge, MA, USA) was employed.

### Western blots

Frozen tissues were lysed in RIPA lysis buffer (150 mM NaCl, 50 mM Tris, pH 7.4, 1 mM EDTA, 1% Triton X-100 or 1% NP40, 10% glycerol, 1 mM phenylmethylsulfonyl fluoride, Na_3_VO_4_ and protease inhibitor cocktail). Lysates were mixed with 4× NuPAGE LDS loading buffer and dithiothreitol, and 50 μg protein was loaded per lane on a NuPAGE 4-12% Bis-Tris gradient gel and transferred to polyvinylidene difluoride membrane (run at 100 V for 1.5 h). Membranes were transferred to blocking buffer for 1 h at room temperature and then probed with primary antibodies: rabbit polyclonal anti-TrkB (1:3000; 07-225, Upstate USA); rabbit polyclonal anti-phospho-TrkB^Y817^ (1:10,000; 2149-1, Epitomics, Burlingame, CA, USA); mouse monoclonal anti-phospho-ERK^T202/Y204^ (1:2000; 9106), rabbit polyclonal anti-ERK (1:2000, 9101), mouse monoclonal anti-phospho-AKT^S473^ (1:2000; 4051), rabbit polyclonal anti-AKT (1:2000; 9272) (all from Cell Signaling Technology); and mouse monoclonal anti-actin (1:10000; A5441, Sigma-Aldrich). For phospho-protein detection, blots were probed with the antibody for each phospho-protein added either in a separate hybridization step, or with two antibodies present in tandem as indicated in the Fig. 4 legend. The same blots were stripped and re-probed with antibodies directed against total TrkB, AKT and ERK. Because TrkB, AKT and ERK proteins run at distinct molecular masses, total antibodies for all three proteins were added at the same time to the probing solution to detect the three respective bands in each blot. Primary antibody incubations were overnight at 4^o^C followed by incubation with the appropriate HRP-conjugated secondary antibody for 1 h at room temperature. The bands were developed by incubating membranes in ECL developing solutions mixed in equal volumes for 1 min, followed by imaging with Kodak film.

### Brain:plasma partitioning studies

Brain and plasma concentrations of LM22A-4, PTX-BD4-3 and atenolol were determined by LC-MS/MS by Absorption Systems (Exton, PA, USA). Male C57BL/6 were dosed intraperitoneally one time with LM22A-4 or PTX-BD4-3 suspended in water at 50 mg/kg (10 ml/kg). Mice were sacrificed at 1 h or 3 h post dose (*n*=3-4 mice/time point), and brain and plasma samples were collected. One animal was excluded from analysis because it exhibited compound levels that were more than 3 standard deviations about the population mean, suggesting a possible dosing error.

Cerep screen (radioligand binding assay) and ADME-Tox

To evaluate the relative binding specificity of LM22A-4 (Massa et al., 2010) and PTX-BD4-3, binding to a broad range of 55 pharmacologically relevant receptors, including numerous neurotransmitter and G-protein coupled receptors was assessed using standard radioligand competition binding assays (ExpressSProfile) performed by Cerep, Inc. (Seattle, WA, USA). In addition, ADME-Tox analysis was performed by Cerep, Inc. All experimental conditions are described on the Cerep website (www.cerep.fr).

### *In vivo* drug treatments

Small-molecule compounds were diluted in saline and delivered by i.p. injection. To define the acute effects of single drug treatments, behavioral testing was initiated 1 h after dosing. Durable effects of single drug treatments were evaluated 24 h after dosing. To define the effects of repeated dosing, mice were treated once every 3 days (q72 h) for 4 or 8 weeks, and behavioral testing was performed 24 h after the last dose.

### Randomization and blinding

Prior to each experiment, animals were numerically coded and assigned to treatment groups by computerized random number selection. Drug and saline injections, behavioral testing and data analysis were all performed by investigators blinded to genotype and treatment.

### Whole-body plethysmography

Breathing was recorded in unrestrained Wt and Het mice using a whole-body plethysmograph (EMMS Systems), in which a constant bias flow supply connected to the animal recording chamber ensured continuous inflow of fresh air (1 l/min) and ambient temperature was maintained between 23°C and 25°C. Animals were allowed to acclimate in the recording chambers for 30 min, and respiratory data were collected for the next 3 h. Because respiration is strongly influenced by behavioral state, data were only analyzed from periods of quiet breathing, which was defined as periods when the animal was not moving and had all four paws on the chamber floor. In most experiments, aggregated episodes of quiet breathing totaling at least 10 min were used for analysis. In some experiments in which insufficient numbers of animals exhibited ≥10 min of quiet breathing, aggregated episodes totaling at least 2 min were used. Apneas were defined as breathing pauses longer than 2× the average total breath duration (Ttot) measured during quiet breathing and are reported here as apneas/min (apnea index). Het mice typically exhibit an apnea index that is ∼2-fold higher than in Wt animals. Other respiratory parameters, such as breathing frequency, were not significantly different between Wt and Het animals in these experiments.

### Foot slip

Foot slip was used to assess locomotor coordination and spontaneous movement on a challenging substrate. Animals were placed on an elevated wire mesh surface (50 cm^2^) with 1 cm^2^ openings and allowed to freely explore for 5 min (Starkey et al., 2005). Total distance traveled was measured by a computer-operated animal activity system (ANY-maze, Stoelting). The total number of forepaw and hindpaw slips, where the foot came completely through the grate with no toes remaining on the grid, was manually counted, and the number of slips/cm traveled was calculated.

### Rotarod

A Rotarod apparatus (Columbus Instruments) was used test to assess sensorimotor coordination. Animals were placed on a rotating rod at a constant speed of 4 rpm for 1 min of acclimation. After the acclimation period, the speed gradually increased (0.1 rpm/s) until the animal fell off of the rod or hung on for a full rotation. Latency of the animal to fall was recorded. The test was repeated three times, with each trial separated by 15 min, to assess potential learning components of any observed phenotypes.

### Statistical analyses

For the *in vitro* studies, all analyses were conducted blind to the experimental conditions. Statistical significance was determined using one-way analysis of variance (ANOVA) with Dunnett's multiple comparison post hoc testing with SPSS software. In cases in which data did not follow a normal distribution, a Mann–Whitney or Kruskal–Wallis test (non-parametric ANOVA) with Dunn's multiple comparison test was applied. Results are expressed as group mean±s.e.m. and statistical significance was set at *P*≤0.05. For the *in vivo* studies, behavioral data were analyzed by one-way ANOVA followed by post hoc analysis with least squares difference (LSD) or Bonferroni tests. Pharmacokinetic comparisons between PTX-BD4-3 and LM22A-4 were analyzed by one-tailed Student's *t*-test, as appropriate for testing the hypothesis that modification of the parent compound would impact the pharmacokinetic profile in a single direction, e.g. to increase the brain-to-plasma ratio. With the exception of the one extreme outlier described in the blood-brain partitioning studies, no data were excluded from any other analyses.

## Supplementary Material

Supplementary information
