# Supplementary material for: Restoration of motor learning in a mouse model of Rett syndrome following long-term treatment with a novel small-molecule activator of TrkB
Source: Dis Model Mech. 2020 Nov 27;13(11):dmm044685. doi: 10.1242/dmm.044685 (PMC7710018; doi:10.1242/dmm.044685)
Supplement: Supplementary information [file dmm-13-044685-s1.pdf]

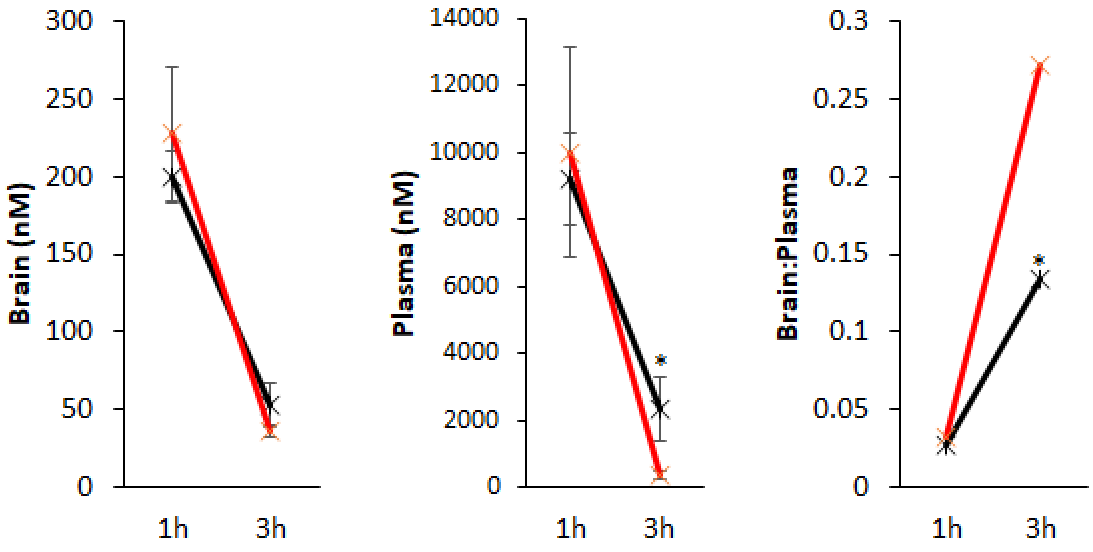

Figure S1. Brain and plasma levels, and brain to plasma ratios for LM22A-4 and PTX-BD4-3 following IP administration in Wt mice. Brain and plasma levels were determined by LC-MS/MS and used to calculate the brain:plasma ratios at 1 and 3 hours after IP administration of 50 mg/kg LM22A-4 (black), or PTX-BD4-3 (red), in mice (n=11-12 mice/time point; data shown are mean ± s.e.m.) *p<0.05 compared to LM22A-4 at same time point; one-tailed Student's t-test.

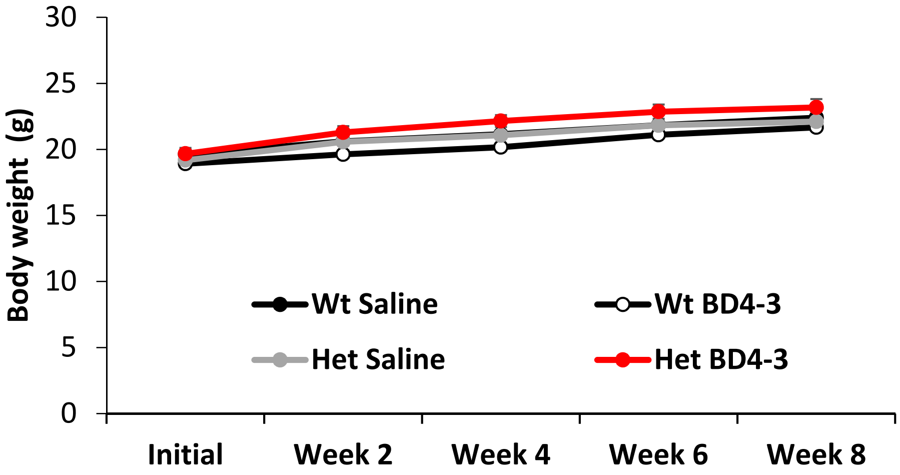

Figure S2. Repeated dosing with PTX-BD4-3 (5 mg/kg, q72 hrs) had no effect on body weight in either Wt or Het mice.

Disease Models & Mechanisms • Supplementary information

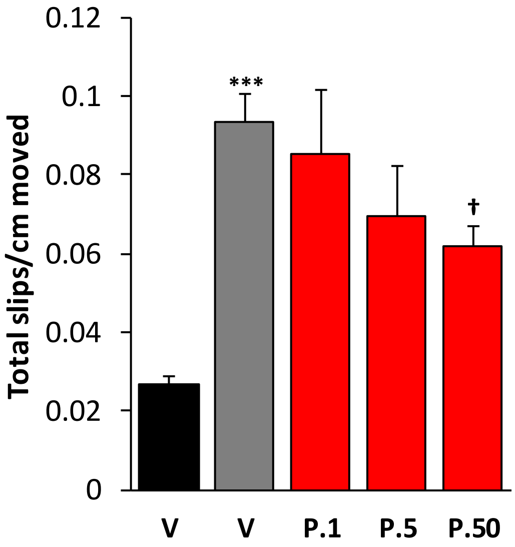

Figure S3. Single administration of PTX-BD4-3 reduces foot slip errors in Het mice. Mice received a single injection of PTX-BD4-3 (P.1, P.5, P.50) one hour prior to the beginning of foot slip testing. n = 10-23 animals derived from 3 independent experiments. ***$p<0.001$ compared to saline-treated Wt mice (V, black bar), †$p<0.05$ compared to saline-treated Het mice (V, grey bar); One-Way ANOVA with LSD post-hoc analysis.

	Ligand	% Inhibition of Control Specific Binding		
		1st	2nd	Mean
1	A_1 *(h)* (agonist radioligand)	8.9	5.6	7.2
2	A_{2A} *(h)* (agonist radioligand)	11.4	30.1	20.7
3	A_3 *(h)* (agonist radioligand)	-1	-9.7	-5.4
4	α_1 (non-selective) (antagonist radioligand)	0.9	-4.4	-1.7
5	α_2 (non-selective) (antagonist radioligand)	1.8	4.7	3.2
6	β_1 *(h)* (agonist radioligand)	3.7	-11.4	-3.9
7	β_2 *(h)* (agonist radioligand)	-2.2	-8.2	-5.2
8	AT_1 *(h)* (antagonist radioligand)	-1.6	-49.3	-25.4
9	BZD (central) (agonist radioligand)	2.9	-4.4	-0.7
10	B_2 *(h)* (agonist radioligand)	8.1	0.3	4.2
11	CB_1 *(h)* (agonist radioligand)	-2	-1.4	-1.7
12	CCK_1 (CCK_A) *(h)* (agonist radioligand)	-8.6	-5.1	-6.8
13	D_1 *(h)* (antagonist radioligand)	10.7	1.3	6
14	D_{2S} *(h)* (antagonist radioligand)	-27.9	2.4	-12.7
15	ET_A *(h)* (agonist radioligand	-12.1	-1.1	-6.6
16	GABA (non-selective) (agonist radioligand)	16.5	19.1	17.8
17	GAL_2 *(h)* (agonist radioligand)	-10.8	6.3	-2.2
18	CXCR2 (IL-8B) *(h)* (agonist radioligand)	-6.9	-12.6	-9.7
19	CCR1 *(h)* (agonist radioligand)	-26.1	-15.1	-20.6
20	H_1 *(h)* (antagonist radioligand)	5.8	5.2	5.5
21	H_2 *(h)* (antagonist radioligand)	2.8	-17.3	-7.3
22	MC_4 *(h)* (agonist radioligand)	-31.9	-43.2	-37.6
23	MT_1 (ML_{1A}) *(h)* (agonist radioligand)	-15.4	-21.1	-18.3
24	M_1 *(h)* (antagonist radioligand)	5	15	10
25	M_2 *(h)* (antagonist radioligand)	6.3	-7.9	-0.8
26	M_3 *(h)* (antagonist radioligand)	-22.1	0.8	-10.7
27	NK_2 *(h)* (agonist radioligand)	-25.4	-3.6	-14.5
28	NK_3 *(h)* (antagonist radioligand)	-12.2	0.4	-5.9
29	Y_1 *(h)* (agonist radioligand)	-7.7	-3.2	-5.4
30	Y_2 *(h)* (agonist radioligand)	-5.4	-25	-15.2
31	NTS_1 (NT_1) *(h)* (agonist radioligand)	11.2	2.6	6.9
32	δ_2 (DOP) *(h)* (agonist radioligand)	-6	1.9	-2
33	κ (KOP) (agonist radioligand)	0.9	-1.6	-0.3
34	μ (MOP) *(h)* (agonist radioligand)	6.6	-9.5	-1.4
35	NOP (ORL1) *(h)* (agonist radioligand)	-6.1	6.3	0.1
36	EP_4 *(h)* (agonist radioligand)	9.5	10.4	9.9
37	$5\text{-}HT_{1A}$ *(h)* (agonist radioligand)	19.3	-9.2	5
38	$5\text{-}HT_{1B}$ (antagonist radioligand)	-4.1	-6.2	-5.1
39	$5\text{-}HT_{2A}$ *(h)* (antagonist radioligand)	4.8	-4.8	0

Disease Models & Mechanisms • Supplementary information

40	5-HT_{2B} *(h)* (agonist radioligand)	12.6	-5.6	3.5
41	5-HT_3 *(h)* (antagonist radioligand)	6.9	13.4	10.1
42	5-HT_{5a} *(h)* (agonist radioligand)	-9.2	-15.6	-12.4
43	5-HT_6 *(h)* (agonist radioligand)	-11.7	-7.7	-9.7
44	5-HT_7 *(h)* (agonist radioligand)	8.1	14.7	11.4
45	sst (non-selective) (agonist radioligand)	5.7	-5.2	0.3
46	$VPAC_1$ (VIP_1) *(h)* (agonist radioligand)	-12.3	-10.1	-11.2
47	V_{1a} *(h)* (agonist radioligand)	-12.4	-0.5	-6.5
48	Ca^{2+} channel (L, verapamil site) (phenylalkylamine) (antagonist radioligand)	0	-1.3	-0.7
49	K_V channel (antagonist radioligand)	6.4	-7.9	-0.7
50	SK_{Ca} channel (antagonist radioligand)	-26.8	-44.4	-35.6
51	Na^+ channel (site 2) (antagonist radioligand)	0.6	11.7	6.1
52	Cl^- channel (GABA-gated) (antagonist radioligand)	2.7	-5.3	-1.3
53	norepinephrine transporter *(h)* (antagonist radioligand)	5.1	1.1	3.1
54	dopamine transporter *(h)* (antagonist radioligand)	2.2	0.9	1.6
55	5-HT transporter *(h)* (antagonist radioligand)	16.3	5.5	10.9

Table S1. *In vitro* binding assays. An ExpresSProfile screen of PTX-BD4-3 binding to a panel of pharmacologically relevant receptors was conducted by Cerep, Inc. (Eurofins Panlabs, MO). The results are expressed as percent inhibition of control specific binding obtained in the presence of 10 micromolar PTX-BD4-3. In this assay, values below 50% indicate no specific binding of PTX-BD4-3. The assay was negative for PTX-BD4-3 binding to all of the 55 receptors in the screen.

Disease Models & Mechanisms • Supplementary information

	Aqueous solubility (µM)	Human hepatocyte metabolic stability (min)	CYP inhibition (10µM)	AMES DNA mutagenesis	hERG testing
**LM22A-4**	180.2	>60	negative	negative	negative
**PTX-BD4-3**	200	>60	negative	negative	negative

Table S2. Solubility, metabolism and toxicity screens for LM22A-4 and PTX-BD4-3. Standardized assays of solubility, metabolic stability and *in vitro* toxicity were performed to further evaluate the potential of PTX-BD4-3 as a lead candidate molecule for the treatment of Rett syndrome, and to compare its profile to that of the parent compound LM22A-4. Aqueous solubility of both compounds, which is used as a surrogate measure of absorption, was at or near the assay maximum of 200 µM. The metabolic stability of both compounds at 10 µM was evaluated at 5 different time points in human liver microsomes and found to be >60 min, indicating favorable stability. Both compounds were negative in 3 different assays of *in vitro* toxicity; inhibition of 8 different CYP enzymes at 10 µM, DNA mutagenesis in 4 different strains of Salmonella typhimurium at 5, 10, 50 and 100 µM and cardiac toxicity, measured by activation of hERG channels at 0.1, 1 and 10 µM, where >50% inhibition of the tail current indicates significant cardiac toxicity.

Disease Models & Mechanisms • Supplementary information

Group	Pre-Treatment	Week 2	Week 4	Week 6	Week 8
Het Saline	2.81	4.43	5.06	4.43	4.50
Het PTX-BD4-3	2.73	3.46	3.66	4.33	3.66

Table S3. Group average bi-weekly phenotype severity scores after repeated dosing with PTX-BD4-3 (5 mg/kg, q72 hrs). Animals were assigned aggregate scores of phenotypic severity based on visual assessments of mobility, hind limb clasping, gait analysis, tremors, respiration and overall physical condition (scoring range = 0-12; Guy et al., 2007). The phenotype severity scores of Het animals treated with saline increased significantly between the pre-treatment period (M=2.8125, SD=0.6551) and the 8 week treatment time point (M=4.5, SD=1.62); t(15) = -4.163, p=0.0008. The phenotype severity scores of Het animals treated with PTX-BD4-3 did not change significantly over the same treatment period; pre-treatment (M=2.7333, SD=1.387); 8 weeks (M=3.6666, SD=1.7182); t(14) = -1.584, p=0.1356.

Disease Models & Mechanisms • Supplementary information
